# 1312. Incidence and Microbiology of Prosthetic Joint Infection According to Time Following Total Knee Arthroplasty: A Retrospective Cohort Study Among US Veterans

**DOI:** 10.1093/ofid/ofad500.1151

**Published:** 2023-11-27

**Authors:** Erica J Weinstein, Vincent Lo Re III, Alisa Stephens-Shields, Craig Newcomb, Randi Silibovsky, Charles Nelson, Judith O’Donnell, Laurel Glaser, Evelyn Hsieh, Jennifer Hanberg, Kathleen Akgün, Janet Tate, Joseph King

**Affiliations:** Perelman School of Medicine at the University of Pennsylvania, Philadelphia, Pennsylvania; University of Pennsylvania, Philadelphia, PA; Perelman School of Medicine at the University of Pennsylvania, Philadelphia, Pennsylvania; Perelman School of Medicine at the University of Pennsylvania, Philadelphia, Pennsylvania; Perelman School of Medicine at the University of Pennsylvania, Philadelphia, Pennsylvania; Perelman School of Medicine at the University of Pennsylvania, Philadelphia, Pennsylvania; Perelman School of Medicine at the University of Pennsylvania, Philadelphia, Pennsylvania; University of Pennsylvania, Philadelphia, PA; Yale University School of Medicine and VA Connecticut Health System, West Haven, Connecticut; Massachusetts General Hospital, Boston, Massachusetts; Yale University School of Medicine and VA Connecticut Health System, West Haven, Connecticut; Yale University School of Medicine and VA Connecticut Health System, West Haven, Connecticut; Yale University School of Medicine and VA Connecticut Health System, West Haven, Connecticut

## Abstract

**Background:**

Despite the frequency of total knee arthroplasty (TKA) and clinical impact of prosthetic joint infections (PJIs), the incidence and microbiology of these infections in the United States remains unclear. Our objective was to determine the incidence of PJI occurring ≤ 3 months (early), between > 3 and ≤ 12 months (delayed), and > 12 months (late) after primary TKA and to identify the organisms isolated from microbiologic cultures during each of these periods.

**Methods:**

We conducted a retrospective study of patients in the Veterans Aging Cohort Study- National Cohort who underwent elective primary TKA in the VA between October 1, 1999 to September 30, 2019. The primary outcome was incident hospitalization with PJI. We estimated incidence rates (events/10,000 person-months) with 95% confidence intervals (CIs) of early, delayed, and late PJI. Poisson regression was used to estimate the incidence rate ratio (IRR) with 95% CI of early and delayed PJI compared to late PJI. The Kaplan-Meier method was used to demonstrate the cumulative incidence of PJI at 3, 12 and 24 months. We measured the frequency of Gram-positive, Gram-negative, fungal, polymicrobial, and culture-negative PJI occurring within each of the three time periods and compared frequencies of interest using Pearson’s chi-squared tests.

**Results:**

Among 82,401 VHA patients who underwent primary TKA, a total of 1,605 incident PJIs (1.9%) were identified. The incidence rate of PJI was higher in the first 12 months after primary TKA surgery, with an IRR of 20.4 (95% CI, 18.2-222.8) for early and 4.2 (95% CI, 3.7-4.8) for delayed periods when compared to late. The cumulative incidence of PJI at 3 months was 0.77% (95% CI, 0.71-0.83%), at 12 months was 1.25% (95% CI 1.17-1.33%), and at 24 months was 1.56% (95% CI 1.48-1.65%) (**Figure 1**). *Staphylococcus aureus* was the most common organism isolated (33.2%) across the periods. Gram-negative bacteria were isolated in 11.1% of all infections, and 15.1% of early PJIs (**Table 1**).

Kaplan Meier failure curves for prosthetic joint infection for the overall study period (upper panel) as well as the first 24 months (lower panel) following primary total knee arthroplasty.

**Figure:**
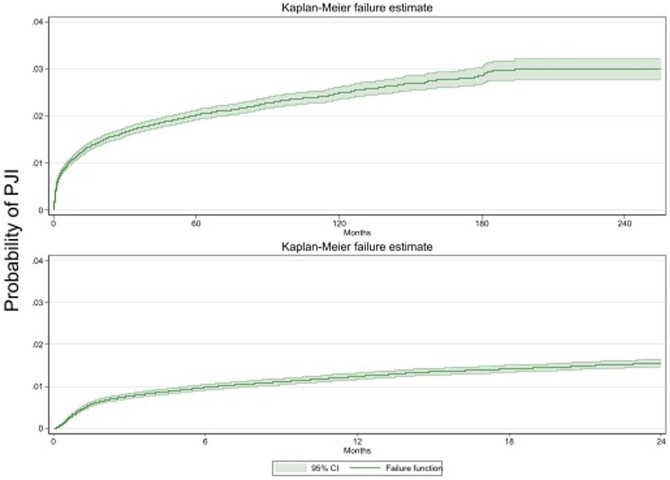

Abbreviations: PJI, prosthetic joint infection; CI, confidence interval.

**Table 1.**
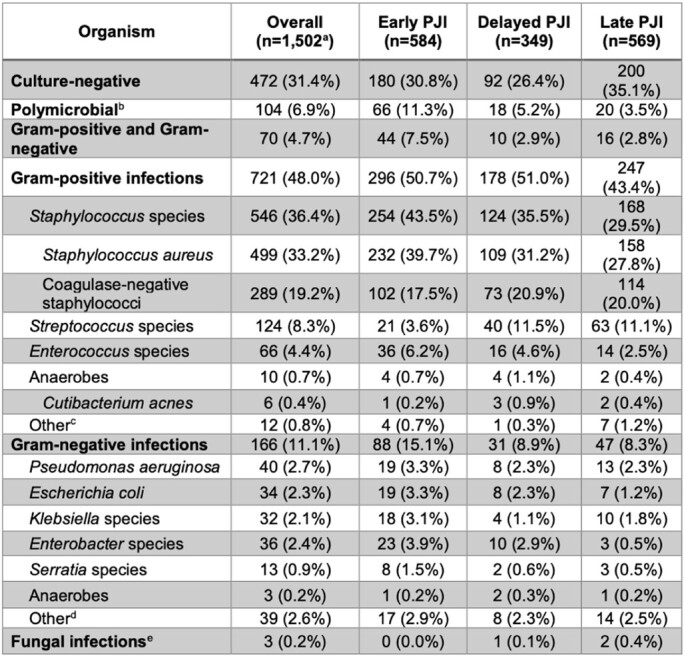
Organisms isolated from synovial fluid or operative tissue cultures of early, delayed and late prosthetic joint infections after primary total knee arthroplasty.

^a^Among the 1,605 PJIs in the Overall Cohort, the microbiology data for 103 were missing and 1,502 PJIs with available microbiology data were included in these results. ^b^Polymicrobial defined by multiple genera of organisms identified ^c^Includes: Micrococcus species, diphtheroids ^d^Includes: Salmonella, Pasteurella, Citrobacter, Morganella, Acinetobacter, Pasteurella, Burkholderia species, and Gram-negative organisms not otherwise specified ^e^All fungal infections were identified as Candida species ^f^Numbers may not sum to group totals or percentages as polymicrobial infections with more than one organism were possible Abbreviations: PJI, prosthetic joint infection.

**Conclusion:**

Incidence rates of early and delayed PJI were significantly higher than late PJI following primary TKA. Gram-negative organisms were more prevalent in early compared to delayed or late PJI. Empiric Gram-negative antibiotic therapy should be considered with a suspected PJI within 3 months after elective primary TKA.

**Disclosures:**

**Alisa Stephens-Shields, PhD**, Gilead Sciences: Advisor/Consultant **Charles Nelson, MD**, Stryker: Advisor/Consultant|Zimmer-Biomet: Advisor/Consultant|Zimmer-Biomet: Grant/Research Support

